# Frequency of low-grade adverse events and quality of life during chemotherapy determine patients’ judgement about treatment in advanced-stage thoracic cancer

**DOI:** 10.1007/s00520-019-4659-x

**Published:** 2019-01-28

**Authors:** Mark de Mol, Sabine Visser, Brenda L. den Oudsten, Paul Lodder, Nico van Walree, Huub Belderbos, Joachim G. Aerts

**Affiliations:** 1 Department of Pulmonary Diseases, Amphia Hospital, P.O. Box 90158, 4800 RK Breda, The Netherlands; 2 Department of Pulmonary Diseases, Erasmus MC Cancer Institute, P.O. Box 2040, 3000 CA Rotterdam, The Netherlands; 3 Department of Epidemiology, Erasmus MC—University Medical Centre Rotterdam, P.O. Box 2040, 3000 CA Rotterdam, The Netherlands; 4 Department of Medical and Clinical Psychology, Centre of Research on Psychological and Somatic Disorders (CoRPS), Tilburg University, P.O. Box 90151, 5000 LE Tilburg, The Netherlands; 5 Department of Methodology and Statistics, Tilburg University, P.O. Box 90151, 5000 LE Tilburg, The Netherlands

**Keywords:** Cancer Therapy Satisfaction Questionnaire, Psycho-oncology, Adverse events, Anti-neoplastic therapy, Non-small cell lung cancer

## Abstract

**Purpose:**

In lung cancer, the preservation of well-being is warranted given the limited prognosis. Chemotherapy may negatively influence health-related quality of life (HRQoL) due to adverse events. However, patients’ judgement about this negative impact is not well understood. We examined the relationship between expectations, feelings about side effects, and satisfaction with therapy and (HR)QoL in advanced-stage thoracic cancer and investigated which of these factors has the highest impact on (HR)QoL.

**Methods:**

Sixty-nine patients completed the Cancer Therapy Satisfaction Questionnaire (CTSQ), the World Health Organization Quality of Life-BREF (WHOQOL-BREF), and the European Organization for Research and Treatment of Cancer Quality of Life Questionnaire-Core 30 (EORTC QLQ-C30)*.* Multiple regression analyses were performed to investigate the relation of the CTSQ domains (i.e., expectations of therapy, feelings about side effects, satisfaction with therapy) with (HR)QoL and simple regression analyses to identify the factors of the CTSQ domain that was most often associated with (HR)QoL.

**Results:**

Feelings about side effects were associated with the (HR)QoL domain/scale scores (i.e., WHOQOL-BREF domains: *β* = 0.36 to 0.58; EORTC QLQ-C30 scales: *β* = 0.33 to 0.61) except social relationships of the WHOQOL-BREF. Low-grade adverse events were related to feelings about side effects (*β* = − 0.326; *P* = 0.007).

**Conclusions:**

Patients experiencing negative feelings about side effects have worse (HR)QoL. Additional care should be provided to prevent low-grade adverse events.

## Introduction

In patients with advanced-stage lung cancer, the preservation of their well-being is warranted given their, in general, limited prognosis [1, 2]. Chemotherapy may have negative impact on patients’ health-related quality of life (HRQoL) due to side effects [3]. However, it is not well understood what aspect of chemotherapy causes this potential negative effect on QoL. The Cancer Therapy Satisfaction Questionnaire (CTSQ) is an instrument that assesses patients’ expectations, their feelings about side effects, and their satisfaction with therapy. Application of this questionnaire gives more insight in patients’ view on treatment.

Although several publications reported about patients’ satisfaction with care [4–6], patients’ opinions related to side effects were not evaluated in these studies. Moreover, in a study by Rha et al., it was observed that clinicians underestimated the impact of side effects compared to patients. In addition, physicians rated different symptoms (i.e., nausea and vomiting) as most problematic than patients (i.e., fatigue and anorexia) did [7]. The CTSQ assesses the feelings patients have about treatment [8]. As such, the CTSQ could inform physicians about patients’ treatment-related opinions, which may facilitate the management of (HR)QoL. For instance, if a patient scores low on the feelings about side effects domain of the CTSQ, this is a clear indicator that they are bothered by side effects. Subsequent identification and adequate management of the experienced side effects may offer opportunities to maintain (HR)QoL at an acceptable level.

However, the CTSQ may also be useful in the process of clinical decision making. In many patients with advanced cancer, a physician’s decision to start with treatment is related to a patient’s functional status, comorbidity, and potential toxicity [9, 10], whilst patients often focus on survival benefits [10, 11] and may accept a decrease in QoL [12]. Moreover, patients with cancer would like to be involved in treatment decisions [13]. A considerable proportion (38.3%; *n* = 49) of patients with lung cancer preferred to have some input in treatment decision making or would like shared treatment decisions. However, this was achieved in only 46.9% (*n* = 23) of the 49 cases [14]. Therefore, exploring a patient’s treatment-related opinion is important as they could have a different understanding of survival rates and the impact of side effects on (HR)QoL than their physicians.

In previous studies, we and others have shown that the domains of the CTSQ (i.e., expectations of therapy, feelings about side effects, satisfaction with therapy) are related to (HR)QoL [15, 16]. In this study, we investigate which of the CTSQ domains are associated with (HR)QoL at the end of treatment in patients with advanced-stage lung cancer and mesothelioma. In addition, we assess which underlying factors (i.e., sociodemographic and clinical variables) are associated with the CTSQ domain that is most often significantly related with (HR)QoL.

## Methods

### Study population

PERSONAL is a prospective observational multi-center cohort study of patients with locally advanced or metastatic (i.e., stage IIIB or IV) non-squamous non-small cell lung carcinoma (NSCLC) and unresectable mesothelioma treated with pemetrexed. Patients were recruited from October 2012 to November 2014 from three teaching hospitals (i.e., Erasmus University Medical Center, Amphia Hospital, and Sint Franciscus Gasthuis Hospital) and a regional hospital (i.e., Bravis Hospital). Patients were enrolled if they met the following criteria: they were aged 18 years or older, had a cytological or histological confirmed diagnosis of advanced or metastatic (i.e., stage IIIB and IV) NSCLC or unresectable malignant pleural mesothelioma, and were treated with at least 4 cycles of pemetrexed in combination with a platinum compound as first-line therapy or with at least 4 cycles of pemetrexed monotherapy as second line. Patients were excluded if they were not able to read Dutch or could not complete the questionnaires due to a physical or mental condition. Informed consent was obtained from all individual participants included in the study. All procedures were in accordance with the ethical standards of the institutional review board of the Erasmus University Medical Center in Rotterdam, The Netherlands (approval number MEC-2012-232), and with the 1964 Helsinki declaration and its later amendments or comparable ethical standards.

### Procedures

The WHOQOL-BREF and EORTC QLQ-C30 were completed by patients before the first cycle of chemotherapy, after the second (days 7 to 14), and after the fourth cycle (days 14 to 21). The CTSQ was completed by patients after the fourth cycle of chemotherapy simultaneously with the (HR)QoL questionnaires. In addition, we collected sociodemographic information (i.e., age, sex, educational level, ethnicity, employment, partner status (i.e., living or not living together with a partner)) and clinical information (i.e., Eastern Cooperative Oncology Group (ECOG) performance status and cancer stage, type of tumor, line of therapy, and tumor response). In the four weeks before completion of the CTSQ, the severity and number of different chemotherapy-related clinical adverse events that patients experienced were assessed at a weekly basis according to Common Terminology Criteria for Adverse Events (CTCAE) version 3.0. The information regarding these adverse events was collected directly from patients during patient interviews and from medical records in the hospital information system.

### Study measures

The CTSQ contains three domains covering 16 items: expectations of therapy (five items), feelings about side effects (four items), and satisfaction with therapy (seven items) [8, 15]. Each item is scored on a Likert scale from 1 (worst response) to 5 (best response). Four items are reverse coded. Domain scores range from 0 to 100, with a higher score representing a better outcome. All patients completed the Dutch translation of the original English CTSQ [16]. Previous studies have assessed the psychometric properties in patients with different forms of cancer, including advanced-stage lung cancer, and demonstrated good results [15, 16].

The WHOQOL-BREF [17, 18] is a short version of the original WHOQOL-100 [19, 20]. It consists of a general facet (two items) and four domains that represent physical health (seven items), psychological health (six items), social relationships (three items), and environment (eight items). Each item is scored on a Likert scale from 1 (worst response) to 5 (best response). Domains of the WHOQOL-BREF are scored on a 4–20 scale and the general facet on a 2–10 scale with higher scores indicating a better quality of life [17]. Previous studies have demonstrated satisfactory psychometric properties of the WHOQOL-BREF in patients with lung cancer [21] and in patients with chronic diseases or different forms of cancer [18] except for the social relationships domain [18, 21].

The EORTC QLQ-C30 is a cancer-specific HRQoL instrument with demonstrated psychometric properties [22] and was originally developed with lung cancer patients [23]. It consists of 30 items and incorporates a global health status/QoL scale, five functional scales, and a number of items assessing additional symptoms or problems. The functional scales represent physical functioning (five items), cognitive functioning (two items), emotional functioning (four items), role functioning (two items), and social functioning (two items). Each of the EORTC QLQ-C30 domains is scored on a 0–100 scale, with higher scores on the functional scales being indicative of better HRQoL, whereas higher scores on the symptom scales are reflective of worse symptoms [23].

### Statistics

Patient characteristics were analyzed with descriptive statistics. Fisher’s exact test was used to compare patients that completed the CTSQ and (HR)QoL questionnaires with those that did not on a selection of categorical clinical and sociodemographic variables. For the variables “age” and “grade 1 or 2 chemotherapy-related clinical adverse events,” the independent *t* test was used. The Mann-Whitney *U* test was used for the variable “grade 3 or 4 chemotherapy-related clinical adverse events”.

Multiple linear regression analyses were performed to identify the relationship between expectations of therapy, feelings about side effects, and satisfaction with therapy with (HR)QoL without prior simple linear regression analyses given the low number of independent variables. As no specific data has been reported in lung cancer, we expected each potential factor to show a medium effect size. According to Cohen, a correlation of 0.3 (or R^2^ = 0.09) constitutes a medium effect [24]. Thus, given an effect size of R^2^ = 0.09, a power of 0.80, and an alpha of 0.05, 69 patients were needed for our main analyses.

Subsequently, simple linear regression analyses were performed to assess the relationship between sociodemographic (i.e., age, sex, ethnicity, education, employment, partner status) and clinical variables (i.e., type of tumor, ECOG performance status, cancer stage, and treatment response) and expectations of therapy, feelings about side effects, or satisfaction with therapy. Regression analyses were performed only on the independent variable (i.e., expectations of therapy, feelings about side effects, or satisfaction with therapy) that was most often significantly associated with (HR)QoL in the previous multiple regression analyses*.*

A P value of 0.05 or lower was considered to be statistically significant. All analyses were performed with IBM SPSS Statistics for Windows version 21.0.

## Results

### Patient selection and characteristics

**Fig. 1:**
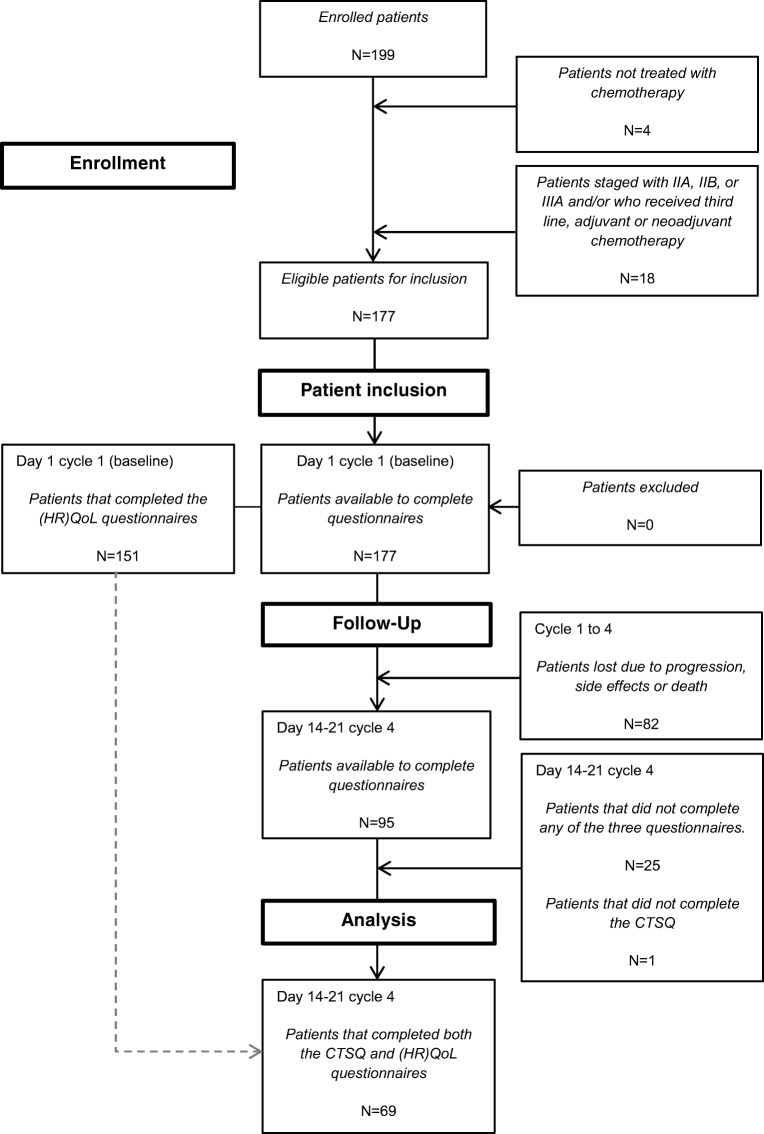
Selection of patients. N number of patients, CTSQ Cancer Therapy Satisfaction Questionnaire, (HR)QoL (health-related) quality of life

Of the 177 patients eligible for inclusion, 95 patients (54%) with stage IIIB or IV NSCLC or mesothelioma completed all 4 cycles of chemotherapy (Fig. 1). Twenty-six of these patients (26%) did not complete the (HR)QoL questionnaires and/or the CTSQ. These patients did not differ with the 69 patients (73%) according to age, sex, ethnicity, education, employment, partner status, cancer stage, type of tumor, line of therapy, ECOG performance status, and number of different grade 1 or 2 or grade 3 or 4 adverse events. Table 1 summarizes the characteristics of all 177 patients and the 69 patients used for the analyses.

**Table 1:** Patient characteristics

Characteristic	All patients (*n* = 177)	Patients that completed all questionnaires (*n* = 69)	Patients that did not complete (all) questionnaires (*n* = 26)	*P* value*
Age, years				
Mean (SD)	63.5 (9.0)	62.7 (8.0)	64.4 (9.8)	0.38
Min, max	37, 83	45, 79	37, 78	
Sex				
Male Female	94 (53.1) 83 (46.9)	38 (55.1) 31 (44.9)	13 (50.0) 13 (50.0)	0.82
Ethnicity				
Caucasian	167 (94.4)	64 (92.8)	24 (92.3)	1.00
Other	10 (5.6)	5 (7.2)	2 (7.7)	
Education^a^				
Low	113 (63.8)	51 (73.9)	18 (69.2)	0.75
High	32 (18.1)	13 (18.8)	3 (11.5)	
Unknown	32 (18.1)	5 (7.2)	5 (19.2)	
Employment				
Yes	39 (22.0)	20 (29.0)	4 (15.4)	0.41
No	112 (63.3)	48 (69.6)	17 (65.4)	
Unknown	26 (14.7)	1 (1.4)	5 (19.2)	
Partner status^b^				
Yes	123 (69.5)	59 (85.5)	15 (57.7)	0.18
No	28 (15.8)	9 (13.0)	6 (23.1)	
Unknown	26 (14.7)	1 (1.4)	5 (19.2)	
Cancer stage^c^				
Locally advanced (IIIB)	21 (11.9)	5 (7.2)	2 (7.7)	0.89
Metastatic (IV)	147 (83.1)	60 (87.0)	22 (84.6)	
Other	9 (5.1)	4 (5.8)	2 (7.7)	
Type of tumor^c^				
Adenocarcinoma	160 (90.4)	63 (91.3)	21 (80.8)	0.17
Large cell carcinoma, mesothelioma, other	17 (9.6)	6 (8.7)	5 (19.2)	
Line of therapy				
First line	161 (91.0)	64 (92.8)	24 (92.3)	1.00
Second line	16 (9.0)	5 (7.2)	2 (7.7)	
ECOG performance status				
Grade 0 or 1	155 (87.6)	66 (95.7)	26 (100.0)	0.20
Grade 2 or higher	21 (11.9)	1 (1.4)		
Unknown	1 (0.6)	2 (2.9)		
Grade 1 or 2 chemotherapy-related clinical adverse events				
Mean		9.2 (3.2)	8.5 (4.0)	0.33
Min, max		3, 19	1, 18	
Unknown		1 (1.4)		
Grade 3 or 4 chemotherapy-related clinical adverse events				
Median		0.0	0.0	0.93
Min, max		0, 4	0, 5	
Unknown		1 (1.4)		

Values are given in numbers (percentages) unless stated otherwise

*n* number of patients, *SD* standard deviation, *ECOG* Eastern Cooperative Oncology Group

^a^Low education: persons whose highest level of education is primary education, lower general education, or lower vocational education. High education: persons whose highest level of education is higher general education, higher vocational education, or university

^b^Partner status: living or not living together with a partner

^c^Measured at baseline

^*^P values describe differences observed with Fisher’s exact test for all categorical variables and with the independent *t* test and Mann-Whitney *U* test for the variables “age” and “grade 1 or 2 chemotherapy-related clinical adverse events” and the variable “grade 3 or 4 chemotherapy-related clinical adverse events”, respectively

### CTSQ domain scores

The median score of the expectations of therapy domain was 55.0 (interquartile range (IQR) 38.8) and that of the feelings about side effects domain was 56.3 (IQR 42.2). Satisfaction with therapy had a median score of 82.1 (IQR 17.9).

### (HR)QoL scale and domain scores

Table 2 demonstrates the scores of the different scales and domains of the EORTC QLQ-C30 and WHOQOL-BREF. For the WHOQOL-BREF, mean domain scores of the normally distributed domains were 13.6 (SD 3.1) for physical health and 16.1 (SD 2.1) for environment. Median scores of the non-normally distributed domains were 13.7 (IQR 4.0) and 15.3 (IQR 2.7) for, respectively, psychological health and social relationships. The median score of the general facet was 6.0 (IQR 3.0). Median scores for the different scales of the EORTC QLQ-C30, including the global health status/QoL scale, ranged from 50.0 (IQR 50.0) to 83.3 (IQR 33.3).

**Table 2:** Results of the WHOQOL-BREF and EORTC QLQ-C30

Questionnaires	Number	Min, max	Mean	SD	Median	IQR
*WHOQOL-BREF*						
Overall QoL/general health	69	3.0, 10.0	6.2	1.7	6.0	3.0
Physical health	67	6.9, 20.0	13.6	3.1	13.7	4.1
Psychological health	68	10.0, 18.7	14.1	2.2	13.7	4.0
Social relationships	68	6.7, 20.0	15.5	2.4	15.3	2.7
Environment	67	11.0, 20.0	16.1	2.1	16.3	3.5
*EORTC QLQ-C30*						
Global health status/QoL	67	8.3, 100.0	57.3	24.6	66.7	41.7
Physical functioning	69	13.3, 100.0	65.1	22.4	66.7	33.3
Role functioning	69	0.0, 100.0	53.1	33.9	50.0	50.0
Emotional functioning	68	16.7, 100.0	75.1	21.5	75.0	25.0
Cognitive functioning	68	0.0, 100.0	77.0	24.4	83.3	33.3
Social functioning	67	0.0, 100.0	74.6	26.8	83.3	33.3
CTSQ						
Expectations of therapy	68	15.0, 100,0	58.1	23.8	55.0	38.8
Feelings about side effects	69	12.5, 100	53.7	25.3	56.3	42.2
Satisfaction with therapy	69	42.9, 100	79.6	13.1	82.1	17.9

*WHOQOL-BREF* World Health Organization Quality of Life-BREF Questionnaire, *EORTC QLQ-C30* European Organization for Research and Treatment of Cancer Quality of Life Questionnaire-Core 30, *CTSQ* Cancer Therapy Satisfaction Questionnaire, *SD* standard deviation, *IQR* interquartile range

### Adverse events

Table 3 describes the occurrence of different chemotherapy-related clinical adverse events according to their grade. Fatigue was the most frequently experienced adverse event with 87.0% of patients reporting fatigue followed by nausea (71.0%) and anorexia (63.8%).

**Table 3:** Ten most frequently reported adverse events according to CTCAE 3.0

Adverse events	Number	Grade 1 or 2	Grade 3 or 4
Total	69		
Fatigue	60	53 (76.8)	7 (10.1)
Nausea	49	46 (66,7)	3 (4.3)
Anorexia	44	42 (60.9)	2 (2.9)
Altered taste	38	38 (55.1)	0 (0.0)
Mucositis	34	33 (47.8)	1 (1.4)
Dry skin	30	30 (43.5)	0 (0.0)
Constipation	30	29 (42.0)	1 (1.4)
Neuropathy sensory	25	25 (36.2)	0 (0.0)
Dizziness	24	24 (34.8)	0 (0.0)
Rash	21	21 (30.4)	0 (0.0)

Values are given in numbers (percentages)

*CTCAE* Common Terminology Criteria for Adverse Events

### The association of the CTSQ with (HR)QoL

For all domains and scales of the (HR)QoL questionnaires, except for the WHOQOL-BREF domain social relationships, the feelings about side effects domain was significantly associated with (HR)QoL (Table 4). Positive feelings about side effects were associated with higher (HR)QoL scores, whereas negative feelings about side effects related with lower (HR)QoL. In contrast, high expectations of therapy were only significantly associated with increased psychological health and high satisfaction with therapy with solely increased global health status/quality of life. No other associations between the (HR)QoL questionnaires and the expectations of therapy and satisfaction with therapy domain were found.

**Table 4:** Results of the multiple regression analyses for the WHOQOL-BREF and EORTC QLQ-C30 domains/scales with the CTSQ domains as variables

Variables	Number	*B*	SE	*β*	*P* value	95% CI for B	*R*^2^
WHOQOL-BREF							
Overall QoL/general health							
ET	68	0.010	0.008	0.143	0.199	− 0.005, 0.026	0.258
FSE		0.031	0.008	0.472	**<** 0.001^*^	0.016, 0.046	
SWT		0.003	0.015	0.026	0.824	− 0.027, 0.033	
Physical health							
ET	66	0.017	0.014	0.135	0.217	− 0.010, 0.045	0.309
FSE		0.063	0.014	0.527	< 0.001^*^	0.036, 0.090	
SWT		0.005	0.027	0.022	0.851	− 0.048, 0.059	
Psychological health							
ET	67	0.020	0.009	0.212	0.032^*^	0.002, 0.038	0.439
FSE		0.050	0.009	0.578	< 0.001^*^	0.033, 0.068	
SWT		0.015	0.017	0.091	0.377	− 0.019, 0.050	
Social relationships							
ET	67	0.015	0.013	0.144	0.256	− 0.011, 0.041	0.044
FSE		0.014	0.013	0.141	0.286	− 0.012, 0.039	
SWT		0.002	0.025	0.013	0.925	− 0.048, 0.052	
Environment							
ET	66	0.011	0.011	0.121	0.310	− 0.010, 0.032	0.166
FSE		0.031	0.010	0.364	0.004^*^	0.010, 0.052	
SWT		0.008	0.021	0.052	0.682	− 0.033, 0.050	
EORTC QLQ-C30							
Global health status/quality of life							
ET	66	− 0.018	0.109	− 0.017	0.869	− 0.237, 0.200	0.339
FSE		0.425	0.106	0.442	< 0.001^*^	0.212, 0.637	
SWT		0.478	0.210	0.257	0.026^*^	0.059, 0.898	
Physical functioning							
ET	68	0.154	0.103	0.162	0.142	− 0.053, 0.360	0.275
FSE		0.376	0.101	0.421	< 0.001^*^	0.174, 0.577	
SWT		0.237	0.200	0.137	0.240	− 0.162, 0.635	
Role functioning							
ET	68	0.179	0.147	0.125	0.227	− 0.114, 0.472	0.360
FSE		0.817	0.143	0.607	< 0.001^*^	0.531, 1.102	
SWT		− 0.192	0.283	− 0.074	0.499	− 0.758, 0.373	
Emotional functioning							
ET	67	0.027	0.105	0.030	0.795	− 0.182, 0.237	0.190
FSE		0.347	0.102	0.412	< 0.001^*^	0.144, 0.550	
SWT		0.085	0.201	0.052	0.672	− 0.316, 0.487	
Cognitive functioning							
ET	67	− 0.043	0.126	− 0.041	0.737	− 0.295, 0.209	0.099
FSE		0.315	0.122	0.329	0.012^*^	0.071, 0.559	
SWT		− 0.222	0.242	− 0.120	0.361	− 0.705, 0.260	
Social functioning							
ET	66	0.019	0.135	0.017	0.887	− 0.251, 0.290	0.149
FSE		0.414	0.131	0.395	0.003^*^	0.151, 0.677	
SWT		− 0.061	0.260	− 0.030	0.815	− 0.581, 0.459	

*CTSQ* Cancer Therapy Satisfaction Questionnaire, *WHOQOL-BREF* World Health Organization Quality of Life-BREF Questionnaire, *EORTC QLQ-C30* European Organization for Research and Treatment of Cancer Quality of Life Questionnaire-Core 30, *ET* expectations of therapy, *FSE* feelings about side effects, *SWT* satisfaction with therapy

^*^P values of ≤ 0.05

### Factors associated with feelings about side effects

In the simple regression analyses, only low-grade chemotherapy-related clinical adverse events (i.e., grade 1 or 2 adverse events) were significantly associated with feelings about side effects (*P* < 0.01) (Table 5). No other relationship was observed.

**Table 5:** Results of the simple regression analyses for the CTSQ FSE domain score

	FSE						
	*n*	*B*	SE	*β*	*P* value	95% CI for *B*	*R*^2^
Age	69	− 0.134	0.383	− 0.043	0.728	− 0.899, 0.631	0.002
Sex	69	− 4.968	6.132	− 0.099	0.421	− 17.206, 7.271	0.010
Ethnicity: Caucasian/other	69	− 8.092	11.780	− 0.084	0.494	− 31.606, 15.421	0.007
Type of tumor: adenocarcinoma/other	69	14.368	10.734	0.161	0.185	− 7.058, 35.795	0.026
ECOG performance score: 0 or 1/higher	69	− 23.878	12.787	− 0.222	0.066	− 49.400, 1.644	0.049
Cancer stage: IIIB/IV	69	9.896	9.020	0.133	0.277	− 8.108, 27.899	0.018
Education: low/high	64	0.129	7.730	0.002	0.987	− 15.323, 15.581	0.000
Employment: yes/no	68	8.238	6.659	0.151	0.220	− 5.056, 21.532	0.023
Partner status: yes/no	68	− 6.128	9.025	− 0.083	0.499	− 24.147, 11.890	0.007
Tumor response: complete and partial response/stable or progressive disease	69	5.525	6.466	0.104	0.396	− 7.382, 18.432	0.011
Grade 1 or 2 chemotherapy clinical AEs	68	− 2.543	0.907	− 0.326	0.007^*^	− 4.354, − 0.733	0.107
Grade 3 or 4 chemotherapy clinical AEs	68	1.527	2.984	0.063	0.610	− 4.430, 7.484	0.004

*CTSQ* Cancer Therapy Satisfaction Questionnaire, *FSE* feelings about side effects, *ECOG* Eastern Cooperative Oncology Group, *AE* adverse event

^*^P values ≤ 0.05

## Discussion

Preservation of (HR)QoL is an important goal during chemotherapy considering that patients with advanced-stage lung cancer have a limited prognosis [1, 2]. Therefore, identification of patients at risk for decreases in (HR)QoL due to treatment may offer opportunities for improvement. We observed, using a validated scoring system to determine patients’ judgement about therapy in different domains, that negative feelings about side effects were associated with decreased (HR)QoL. Especially for patients experiencing low-grade adverse events at a regularly basis, this seems important.

Of the three CTSQ domains, expectations of therapy, satisfaction with therapy, and feelings about side effects, the last one was associated with (HR)QoL. In contrast, satisfaction with therapy was only related with the global health state/QoL scale of the EORTC QLQ-C30. A reason for this may be that none of the seven items of the satisfaction with therapy domain except one (i.e., chemotherapy was worth taking even with side effects), refer to adverse events or (HR)QoL. Moreover, patients may associate satisfaction with therapy with treatment response and survival and not with particular aspects of (HR)QoL. Since the feelings about side effects domain was most often related to (HR)QoL, we studied the underlying factors of this domain. It appeared that the number of different grade 1 or 2 chemotherapy-related clinical adverse events was significantly associated. As these were often experienced on a regularly basis over longer periods of time, vigorous management of them is warranted. Therefore, it is recommended that health care providers have high awareness and consequently check the occurrence and impact of low-grade adverse events as our results clearly demonstrate that patients are bothered by them. In contrast, no relation with chemotherapy-related clinical grade 3 or 4 adverse events was found. This may be because high-grade toxicities were much less experienced in this patient cohort and that the study lacked power. In addition, patients completed the CTSQ after four cycles of chemotherapy. Patients that experienced severe complications may have interrupted chemotherapy and were therefore not included.

Earlier, it was found that HRQoL issues were more often discussed between doctors and patients when the EORTC QLQ-C30 was completed by patients than when this was not the case [13]. All participating physicians and 87% of patients were interested in the persistent use of the questionnaire. These results demonstrated the value of questionnaires in oncological practice. However, application of such an instrument does not provide information about what people think and feel about their treatment. Moreover, (HR)QoL instruments are often more extended than the 16 items of the CTSQ and require more time to be completed which hampers their application during clinical practice. Also, simply the registration of adverse events does not provide information about the extent to which patients are bothered by them. Therefore, considering the results of this study, we advocate the use of the four items of the feelings about side effects domain of the CTSQ as this seems more time efficient and patient friendly.

In the present study, feelings about side effects were more often significantly associated with (HR)QoL than satisfaction with therapy. This is an important observation that may be used by physicians and patients when making treatment decisions. Although several reports reported that patients may accept a decrease in QoL or treatment-related toxicity given a possible survival benefit [11, 12], a systematic review demonstrated that most cancer patients (> 50%) in the included studies required moderate survival benefits to make chemotherapy and its risk for toxicity acceptable [25]. Given that, according to our results, patients with negative feelings about side effects could have low (HR)QoL and that prognosis is limited in advanced-stage lung cancer, we propose that the CTSQ results of previously treated patients may be used to help newly diagnosed patients at risk for adverse events (i.e., decreased performance score, significant comorbidity) in making treatment decisions. For instance, if a considerable proportion of patients who received chemotherapy were often hampered by adverse events according to their CTSQ results, newly diagnosed patients with a limited prognosis could take knowledge of these results and make a more considered treatment decision. In such a way, CTSQ results are handled in a similar manor during decision making as response and survival rates.

Satisfaction with therapy was significantly associated with the global health status/QoL scale of the EORTC QLQ-C30 whereas this was not observed for the general facet of the WHOQOL-BREF. It is possible that this observation is merely due to the idiosyncrasies of the data at hand or simply chance. Also, the relatively small number of patients or selection bias may be responsible for this. In addition, patients may consider occurrence and management of adverse events when they evaluate satisfaction (although this is not directly described by the items that form the satisfaction with therapy domain). Given that adverse events can directly affect a patient’s HRQoL, the interest of health care professionals for adverse events could influence the relation of satisfaction with therapy score with the global health status/QoL scale. For instance, adequate management of adverse events may lead to high patient satisfaction with their care. This may result in increased satisfaction with therapy scores. Given that treatment of adverse events could also enhance HRQoL, increased patient satisfaction with care may result in the observation of an association between satisfaction with therapy and global health status/QoL. Expectations of therapy were significantly associated with psychological health. Besides the possibility of related constructs, reasons for this may be related to coping. For instance, in patients with advanced-stage lung cancer, coping capacity three months after baseline was a predictor for HRQoL [26]. Patients with good coping capacity may have high expectations and may value (HR)QoL more positively than those with few coping capabilities. In addition, coping style may also be of influence as patients that demonstrate “a fighting spirit” may report higher expectations than those that have no hope of a good outcome. Moreover, non-acceptance of the diagnosis and/or prognosis could result in a paradoxical expression of high expectations.

Some limitations of this study have to be addressed. First, the included patients were not asked for their motivation to receive chemotherapy, nor was determined which factors could influence patients’ treatment preferences and opinions. This limited us, together with the observational design of this study and the calculation of associations, to investigate causal relationships between the CTSQ and the (HR)QoL questionnaires. As the present study is part of a larger project in which patients’ motivations were not routinely assessed, we could not provide this information. However, a review that evaluated cancer patients’ preferences for adjuvant therapy reported that in addition to treatment benefit and toxicity, personal experience of the treatment and having dependents (e.g., children) were important determinants of patients’ preferences [27]. Acquiring such information is of importance as it may help physicians to plan their communication strategy towards patients and provides opportunities for personalized treatment.

Second, patients treated with less than four cycles of chemotherapy were not included in this study. These patients dropped out due to progression or adverse events. Given that they had to discontinue treatment with chemotherapy earlier than expected, it is possible that they could have valued satisfaction with therapy more often as important. This could have confounded our results and may explain why satisfaction with therapy in our study was not associated with (HR)QoL. However, other observational studies in patients with advanced-stage lung cancer have experienced similar difficulties with patients dropping out during treatment. In addition, we observed consistent findings regarding the associations of the CTSQ domains with (HR)QoL. Therefore, the findings of the present study contribute to the results of the limited number of reports that discussed the relation of patients’ disease and treatment-related opinions with (HR)QoL.

Third, the observed *R* squares of the simple regression analyses for the feelings about side effects domain in Table 5 were relatively small. To demonstrate with reasonable power that the other predictors were truly not a determinant of feelings about side effects domain score would require the inclusion of many more patients. Given that the *R* square of the analysis in which low-grade adverse events were associated with feelings about side effects score was relatively high, suggesting an acceptable power, the result of this analysis remains of importance.

In conclusion, we demonstrated that patients with advanced-stage lung cancer who experience strong negative feelings about side effects have a decreased (HR)QoL. Our findings demonstrate that low-grade adverse events are of importance for patients’ feelings about side effects. Therefore, it is recommended that in clinical practice, physicians facilitate vigorous management of low-grade adverse events to enhance the (HR)QoL of patients. In addition, the observed results may aid physicians and patients in making treatment decisions.
